# Ubiquitin Proteasome Pathway Transcriptome in Epithelial Ovarian Cancer

**DOI:** 10.3390/cancers13112659

**Published:** 2021-05-28

**Authors:** Jerry Vriend, Mark W. Nachtigal

**Affiliations:** 1 Department of Human Anatomy and Cell Science, Rady Faculty of Health Sciences, University of Manitoba, Winnipeg, MB R3E 0J9, Canada; 2 Department of Biochemistry and Medical Genetics, Rady Faculty of Health Sciences, University of Manitoba, Winnipeg, MB R3E 0J9, Canada; Mark.Nachtigal@umanitoba.ca; 3 Department of Obstetrics, Gynecology & Reproductive Sciences, Rady Faculty of Health Sciences, University of Manitoba, Winnipeg, MB R3E 0J9, Canada; 4 CancerCare Manitoba Research Institute, Winnipeg, MB R3E 0V9, Canada

**Keywords:** ovarian cancer, transcriptome, ubiquitin ligase

## Abstract

**Simple Summary:**

In this review, public datasets were mined in an attempt to identify genes that code for proteins of the ubiquitin proteasome system that can be used as therapeutic targets in high-grade serous ovarian cancer. In this study, we found that more than 50 genes coding for ubiquitin ligases and more than 100 for ubiquitin ligase adaptors were differentially expressed between the low malignant potential tumors and the malignant invasive ovarian tumors. We conclude that several genes coding for the ubiquitin ligases and their adaptors have a potential to serve as therapeutic targets in high-grade serous ovarian cancer.

**Abstract:**

In this article, we reviewed the transcription of genes coding for components of the ubiquitin proteasome pathway in publicly available datasets of epithelial ovarian cancer (EOC). KEGG analysis was used to identify the major pathways distinguishing EOC of low malignant potential (LMP) from invasive high-grade serous ovarian carcinomas (HGSOC), and to identify the components of the ubiquitin proteasome system that contributed to these pathways. We identified elevated transcription of several genes encoding ubiquitin conjugases associated with HGSOC. Fifty-eight genes coding for ubiquitin ligases and more than 100 genes encoding ubiquitin ligase adaptors that were differentially expressed between LMP and HGSOC were also identified. Many differentially expressed genes encoding E3 ligase adaptors were Cullin Ring Ligase (CRL) adaptors, and 64 of them belonged to the Cullin 4 DCX/DWD family of CRLs. The data suggest that CRLs play a role in HGSOC and that some of these proteins may be novel therapeutic targets. Differential expression of genes encoding deubiquitinases and proteasome subunits was also noted.

## 1. Introduction

Gene expression of epithelial ovarian cancers (EOCs) has been studied in terms of the major histological subgroups as well as classifications based on clinical outcome. These studies generated large datasets which included gene expression data that went well beyond the objectives of the original studies. One study of EOC that provided clinical as well as molecular subtypes was the study of Tothill et al. (2008) [1]. In the original Tothill manuscript, six molecular subtypes of ovarian cancer (C1–C6) were described based on gene expression [1], four of which were high-grade serous ovarian cancer (HGSOC) tumors. The Tothill molecular subtypes are not found consistently. Two publicly available (see methods) datasets are the Anglesio dataset [2] and the Bowtell dataset [1]. The Anglesio dataset reports gene expression in low malignant potential (LMP) vs. a HGSOC invasive (INV) group of tumors; the Bowtell dataset reports gene expression in LMP serous tumors vs. malignant (MAL) ovarian tumors that include histotypes other than HGSOC. In this study, we focused primarily on the Anglesio dataset in order to study differentially expressed genes between LMP and HGSOC.

**Figure 1:**
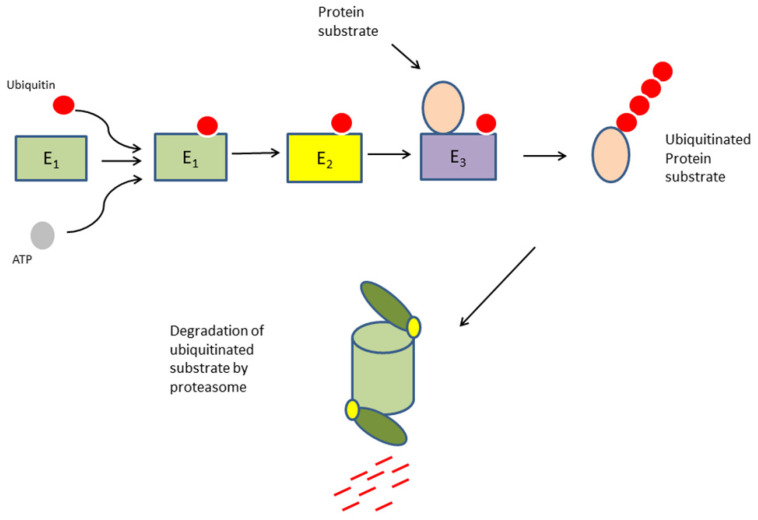
Components of the ubiquitin proteasome pathway in protein degradation.

The ubiquitin proteasome system plays a role in regulating proteins which are risk factors in EOC [3,4,5]. Ubiquitin is added to proteins via three steps involving a ubiquitin activating enzyme (E1), a ubiquitin conjugase (E2), and a ubiquitin ligase (E3) (Figure 1). Ubiquitination of proteins provides a signal for various cellular processes including degradation by the proteasome, regulation of the cell cycle, and modulation of transcription. Ubiquitination may be reversed by deubiqutinases.

Herein, we reviewed the gene expression of ubiquitin conjugases, ubiquitin ligases, ubiquitin ligase adaptors, and deubiquitinases in the publicly available EOC expression datasets comparing LMP and HGSOC and relate the data to the available literature. We also reviewed the data for the gene expression of 43 proteasome subunits. We related the expression of these genes to known pathways in EOC, compared gene expression between LMP and HGSOC, and made suggestions for potential therapeutic targets based on these analyses. We found that the transcription of several ubiquitin conjugases and numerous ubiquitin ligases was different in LMP vs. HGSOC. This is consistent with the view that the mechanism of tumorigenesis and cellular origin may be different between these two groups.

## 2. Materials and Methods

In addition to reviewing the literature on ubiquitin ligases and EOC, we analyzed the relevant publicly available datasets of gene expression in EOC comparing LMP and HGSOC. The expression profiling datasets used are the Anglesio dataset (*N* = 90) [2] and the Bowtell dataset (*N* = 285) [1], both available through the R2 Genomics Analysis and Visualization Platform (R2-GAVP) (http://R2.amc.nl, accessed on 20 January 2021). The data from the Bowtell dataset were reclustered by Anglesio et al. [2] into 2 groups, 32 LMP and 58 HGSOC, referred to as the INV cluster. For the purposes of this review, we will refer to the INV group as HGSOC. Since the malignant group in the Bowtell dataset included histotypes other than HGSOC, in the current study, which is a comparison of LMP and HGSOC gene expression on the ubiquitin proteasome system (UPS), we focused on the Anglesio dataset. However, in several instances, we monitored the Bowtell dataset (LMP vs. MAL) for comparative purposes. The Bowtell data included the 60 HGSOC invasive samples of the Anglesio dataset and 18 of the 30 LMP samples of the Anglesio dataset. The datasets as presented in the R2-GAVP site did not separate the HGSOC data into individual molecular subtypes for either dataset. The Anglesio and Bowtell datasets are also available in the Gene Expression Omnibus site, GSE12172 and GSE9891, respectively.

The R2 genomics site was used to download data for creating heatmaps and for cluster analysis (Morpheus software, Broad Institute, Cambridge, MA, USA, https://software.broadinstitute.org/morpheus, accessed on 20 January 2021) of the LMP and HGSOC tumor subgroups from the Anglesio dataset. The R2-GAVP site was also used for KEGG (Kyoto Encylopedia of Genes and Genomes) pathway analysis. The Ubiquitin and Ubiquitin like Conjugation Database (UUCD) (http://iuucd.biocuckoo.org, accessed on 20 January 2021) was used to identify genes coding for ubiquitin ligases, for ubiquitin ligase adaptors, for ubiquitin conjugases, for deubiquitinases, and for proteasome subunits whose expression was significantly different at *p* < 0.001 in the Anglesio dataset.

## 3. Results and Discussion

### 3.1. KEGG Pathway Analysis Include Ubiquitin Ligase and Ubiquitin Ligase Adaptors

KEGG analysis of the top differentially expressed genes (at *p* < 0.0001) of the Anglesio dataset between the LMP and HGSOC groups shows that the pathways most over-represented were: cell cycle (49 genes), DNA replication (19 genes), p53 signaling (28 genes), Huntington’s disease (54 genes), and the Fanconi anemia pathway (17 genes), in order of statistical significance (Table 1). Table 1 shows the genes in these pathways that encode proteins of the ubiquitin proteasome pathway. These KEGG pathway themes will be integrated as we discuss the role of the ubiquitin proteasome system in serous LMP and HGSOC. Differentially expressed genes of the cell cycle pathway, statistically the major pathway differentiating LMP from the HGSOC group, included *CDC20*. *CDC20*, which codes for a well-known ubiquitin ligase adaptor, is a regulator of the anaphase-promoting ubiquitin ligase complex (APC/c) [6]. Differentially expressed genes of the p53 pathway included *MDM2*, which encodes an E3 ligase that ubiquitinates p53 prior to degradation by the proteasome [7]. Two genes encoding E3 ligase adaptors associated with the dynein motor complex, *DNAI1* and *DNAI2*, represented the Huntington’s disease pathway. Finally, the Fanconi anemia pathway (also known as the FA/Breast Cancer (BRCA) pathway) was represented by expression of the gene encoding the deubiquitinase USP1 and the gene encoding the ubiquitin conjugase UBE2T.

### 3.2. Expression of Genes Coding for Ubiquitin E1 Activators and E2 Conjugases

A modest elevation of expression of the gene encoding the E1 ubiquitin ligase activating enzyme, *UBA1*, was noted in the HGSOC group compared with the LMP group (F = 8.523, *p* < 0.01). UBA1 is one of two enzymes that can activate ubiquitin to begin the ubiquitin cascade [8]. Thus, elevated expression of UBA1 would facilitate altered patterns of ubiquitination in HGSOC cells. While we could find no evidence in the literature that enhanced expression of *UBA1* is associated with EOC progression, UBA1 has been proposed as a therapeutic target for AML [9]. UBA1 inhibitors are available [9,10] and could be investigated in EOC.

Table 2 shows the differential expression of the E2 ubiquitin conjugases. At *p* < 0.0001, the expression of six genes encoding ubiquitin E2 conjugases (*UBE2T, UBE2C, UBE2W, UBE2L6*, *UBE2S*, and *UBE2K*) was higher in the HGSOC group than in the LMP group. Of these genes, the expression of *UBE2T* was statistically the most significant (Table 2). A recent review has related E2 ubiquitin conjugases, including those in Table 2, to various types of disease, including cancer [11].

#### 3.2.1. UBE2T and the Fanconi Anemia/BRCA Pathway

**Figure 2:**
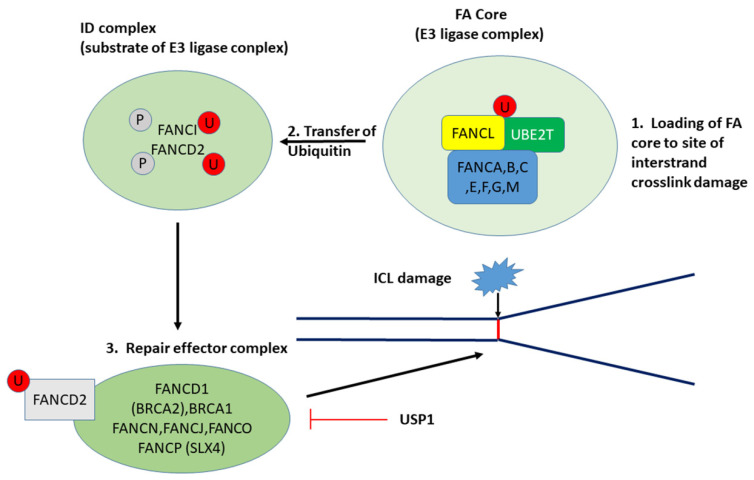
Ubiquitination and deubiquitination in the FA pathway: UBE2T (E2 conjugase), FANCL (E3 ligase), BRCA1 (E3 ligase), and SLX4 (E3 ligase adaptor). U, Ubiquitin; P, phosphate; ICL, interstrand crosslink. USP1 (deubiquitinase). Monoubiquitination of FANCD2 is required for DNA repair. Via UBE2T and FANCL, ubiquitin is transferred to FANCD2. Deubiquitination occurs after DNA repair is completed.

*UBE2T* (also known as *FANCT*) expression appears to be a genetic marker distinguishing LMP from HGSOC (Table 2). It has been reported that increased expression of *UBE2T* is associated with poor survival in EOC [12]. Machida et al. (2006) identified UBE2T as a ubiquitin conjugase essential in the Fanconi anemia pathway (also known as the Fanconi anemia/BRCA pathway) and as a protein that is important in protecting chromosome stability [13]. *UBE2T* has now been well characterized as a gene coding for a ubiquitin conjugase involved in the DNA damage response associated with the Fanconi anemia (FA) pathway [13,14]. *UBE2T* overexpression is associated with several cancers including prostate cancer [15], gastric cancer, [16], breast cancer [17], EOC [12], and multiple myeloma [18]. The role of UBE2T as an E2 conjugase in the FA pathway is to transfer ubiquitin from UBE2T to the E3 ligase FANCL, which, in turn, ubiquitinates FANCD2 (Figure 2) [13,19,20,21]. Ubiquitination of FANCD2 is an indication that the core FA complex is functional [22]. Deubiquitination of FANCD2, in turn, occurs in the nucleus [23] by the deubiquitinase USP1, together with UAF1, after DNA repair is completed [14]. UBE2T also interacts with the BRCA1/BRCA Associate RING Domain 1 (BARD) ubiquitin ligase complex and reportedly ubiquitinates BRCA1 in breast cancer cells [24] and thus, via the BRCA1/BARD1 complex, may be an important contributor to the regulation of genomic stability.

A role of UBE2T in resistance to chemotherapy, as an essential part of the FA pathway, has been reported [21]. UBE2T, as a component of the FA pathway, contributes to the repair of DNA interstrand cross-links [21]. Inhibition of the FA pathway restores DNA cross-link damage associated with chemotherapeutic platinum-based drugs [21,25]. *UBE2T* silencing is reported to inhibit cell proliferation and induce cell cycle arrest in bladder cancer cells [26], lung cancer [27], osteosarcoma [28], and gastric cancer [16]. The data therefore suggest that the identification of a safe and effective UBE2T inhibitor could be a useful adjunct to chemotherapy in HGSOC.

Other members of the FA complementation group of genes include *FANCS* (also known as *BRCA1*); *BRIP* (*FANCJ*), which codes for a BRCA1-interacting protein; and *FANCD1* (also known as *BRCA2*). The HUGO classification lists 22 genes as ‘FA complementation groups’ genes. The expression of 12 of these genes was significantly elevated, at *p* < 0.01, in the HGSOC group (Table 3).

Statistically the most significant of these was *UBE2T* (*p* < 1.0 × 10^−13^). This adds strength to the view that the overexpression of *UBE2T* and its protein may be a major factor in the role the FA pathway plays in HGSOC. It has been shown that resistance to platinum therapeutic agents is related to the FA–BRCA pathway [30]. Additionally, it has been proposed that *FANCF* demethylation results in cisplatin resistance [22]. *FANCF* was the only member of the FA complementation group of genes whose expression was significantly depressed in the HGSOC group. Decreased expression of *FANCF* and its protein has been associated with the reduced ubiquitination of FANCD2 and the increased proliferation of EOC [31,32]. UBE2T plays a key role in the ubiquitination of FANCD2 as part of the core FA complex; it also facilitates the assembly of the core FA complex [33]. Two recent reports described the identification of a UBE2T inhibitor with therapeutic potential [21,34]. The report of Cornwell et al. [21] described a small molecule that specifically inhibited UBE2T/FANCL-induced ubiquitination of FANCD2.

The ubiquitin conjugase UBE2W is also reported to bind to FANCL and regulate FANCD2 [11,35] but its role in the FA pathway is not as well documented as that of UBE2T. Zhang et al. suggested that the mechanism by which UBE2W regulates FANCD2 differs from that of UBE2T [35]. Reportedly, UBE2W, like UBE2T, also binds to the BRCA1–BARD complex and transfers ubiquitin to BRCA1 [36]. The expression of *UBE2W* was elevated in the HGSOC group relative to that of the LMP group (Table 2).

#### 3.2.2. UBE2C, UBE2S, and the Cell Cycle

The ubiquitin conjugase encoded by *UBE2C* (also known as UBCH10) is overexpressed in several cancers, including HGSOC [37,38,39]. UBE2C plays a role in cell cycle progression by interacting with the APC/c complex to initiate the assembly of ubiquitin chains on cell cycle proteins [40]. Subsequently, another ubiquitin conjugase, UBE2S, which also interacts with the APC/c complex, elongates ubiquitin chains [41]. The interaction of UBE2C with APC/c is necessary for mitotic exit [6,42]. A role for UBE2C, and other E2 conjugases, in cancer has been reviewed by Hosseini et al. [11]. These investigators noted that overexpression of UBE2C is associated with poor prognosis in a variety of cancers [11] and promoted the idea of targeting E2 conjugases for cancer therapy. The levels of UBE2C have been reported to be related to the malignancy of EOC and its sensitivity to cisplatin [43]. UBE2C protein has been reported to be upregulated in ovarian cancer and to be a “key protein” in ovarian cancer [44,45,46,47]. In the Anglesio dataset, expression of *UBE2C* was elevated by greater than fiv × 10-fold in the HGSOC group vs. the LMP group (Table 2), and overexpressed by greater than 12-fold compared with the “normal” fallopian tube epithelium, as listed in the Shaw [48] dataset (available in the R2 Genomics Analysis and Visualization Platform). Knockdown of *UBE2C* inhibited the proliferation of EOC cells in culture and reversed resistance to cisplatin [43]. *UBE2S* expression was also elevated in the HGSOC group (Table 2). UBE2S has been reported to promote the proliferation of endometrial cancer cells [49], hepatocellular cancer cells [50], pancreatic cancer [51], and breast cancer cells [52]. The main KEGG pathways associated with *UBE2C* and *UBE2S* expression were cell cycle, DNA replication, oocyte meiosis, and p53 signaling. The data show that the expression of these two genes encoding ubiquitin conjugases is statistically closely associated with the KEGG pathways distinguishing LMP from HGSOC.

*UBE2S* expression was most significantly correlated with *UBE2C* (*r* = 0.76, *p* = 1.83 × 10^−14^) This statistical association was also noted in several datasets of gene expression in breast cancer available in the R2 genomics and visualization site, which included the Bertucci dataset (*n* = 266), the EXPO dataset (*n* = 351), the Yu dataset (*n* = 683), the Miller dataset (*n* = 251), and the Nurses Health Study dataset (*n* = 1110). These data show that there is a strong statistical association between the transcription of these two genes. These remarkable data could be explained by a common regulatory factor stimulating the expression of these two E2 conjugase genes. Their contribution to the ubiquitination of the APC/c RING E3 complex and to cell cycle progression has been extensively studied in breast cancer [52]. Both UBE2S and UBE2C have been proposed as therapeutic targets in various cancers including EOC [52,53].

#### 3.2.3. UBE2L6 and the Immunoproteasome

Expression of *UBE2L6* was also elevated in HGSOC compared with the LMP group (Table 2). *UBE2L6* expression is stimulated by interferon, as is the assembly of the immunoproteasome, a variation of the proteasome found in immune cells [54]. KEGG pathway analysis of gene expression that was significantly correlated with *UBE2L6* in the Anglesio and Bowtell datasets showed various pathways associated with the immune response and T cell-mediated immunity. Gene ontology analysis indicated that the major pathway associated with *UBE2L6* correlations was the Type 1 interferon signaling pathway, consistent with the literature showing that *ULE2L6* expression is stimulated by interferon.

Seifert et al. [54] suggested that, in addition to a role in antigen processing, the immunoproteasome may function to protect cell viability. If this is the case, UBE2L6 and the immunoproteasome may also protect HGSOC cells; the increased expression of *UBE2L6* is consistent with this view. UBE2L6 has been found to be associated with autophagy in esophageal cancer [55] but has not been previously associated with EOC. Murakami et al. [56] found that cisplatin-resistant cervical cancer cells had overexpressed levels of UBE2L6. While we found no association of UBE2L6 with EOC in the literature, the association with cisplatin sensitivity makes it relevant to the treatment of chemoresistant HGSOC.

#### 3.2.4. UBE2F and Neddylation

**Figure 3:**
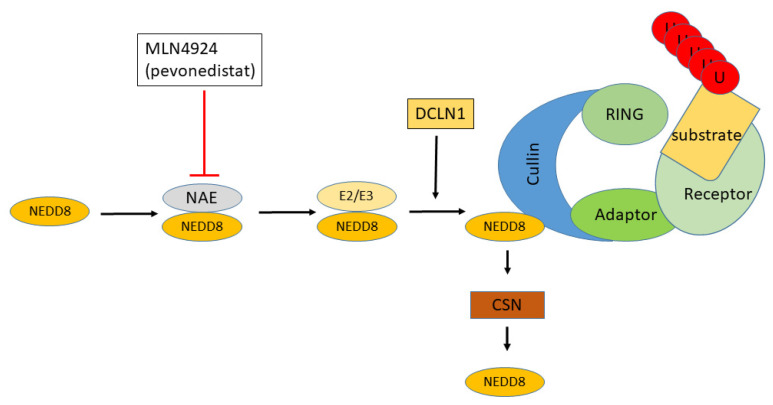
Regulation of Cullin Ring ligases by neddylation. The addition of Nedd8 to CRLs requires E1, NAE, an E2-like Nedd8 conjugase, and an E3 ligase. The CSN signalosome is responsible for deneddylation. Substrates ubiquitinated at K-48 are targeted for destruction by proteasomes. MLN4924 (pevonedistat) is an AMP mimetic that blocks the activation of NEDD8 by NAE.

Neddylation is the addition of NEDD8 to ubiquitin ligases via a three-step process similar to the process of adding ubiquitin to proteins. One of two ubiquitin-like conjugases, UBE2M and UBE2F (also referred to as NEDD8 conjugases), are required to transfer NEDD8 to a NEDD8-specific ligase, which, in turn, transfers NEDD8 to a ubiquitin ligase (Figure 3) [57]. NEDD8 is a protein that activates many E3 ligases [58].

Expression of the genes encoding the NEDD8 conjugases, UBE2M and UBE2K, was significantly greater in HGSOC than in LMP (F = 5.25, *p* < 0.05; F = 22.81, *p* < 0.0001). UBE2M is a NEDD8-conjugating enzyme that facilitates neddylation and activation of Cullin Ring 1–4 ligases, while UBE2F facilitates the neddylation of Cullin Ring 5 ligases [59,60,61]. UBE2F has been promoted as a target to enhance platinum sensitivity in chemotherapy of lung cancer cells [59]. It would be useful to determine whether it is an effective target in EOC.

### 3.3. Ubiquitin Ligases and Adaptors Are Differentially Expressed between LMP and HGSOC

#### 3.3.1. Heatmaps of Expression of E3 Ligases and E3 Adaptors

**Figure 4:**
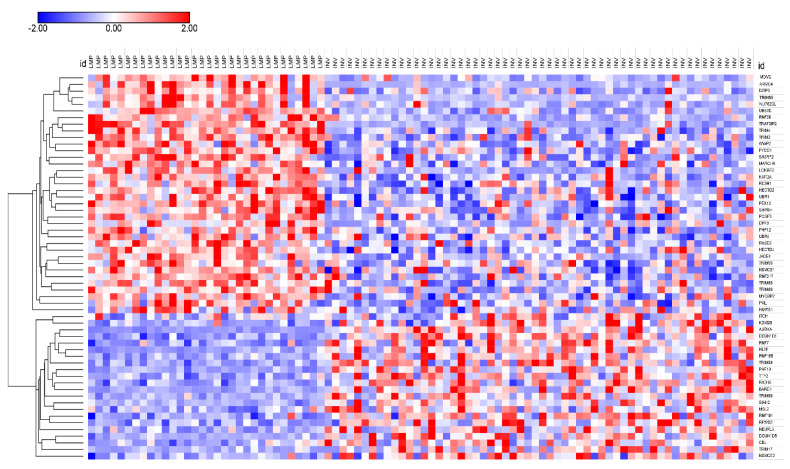
Heatmap and cluster analysis of expression of genes coding for E3 ligases in LMP vs. HGSOC subgroups (data from the R2 Genomics Anglesio dataset, *p* < 0.001, 58 genes).

Numerous genes for E3 ubiquitin ligases were differentially expressed in LMP vs. HGSOC. In the literature, the term E3 ubiquitin ligase usually refers to a complex of proteins including structural proteins, proteins with E3 ligase activity, and proteins with an adaptor/receptor function. The UUCD database distinguishes between genes that code for proteins with E3 ligase catalytic activity and those that code for adaptor proteins. The expression of genes coding for ubiquitin ligases (Figure 4) formed two clusters in the LMP samples, one group whose expression was overexpressed and one that was underexpressed relative to the HGSOC samples. The first cluster included *MDM2*, a gene encoding a ubiquitin ligase involved in the degradation of p53, a gene frequently mutated in HGSOC [7].

The heatmap in Figure 4 illustrates the expression of 58 genes coding for ubiquitin ligases, which were differentially expressed at *p* < 0.001. Of these, 46 were RING (Really Interesting New Gene) ligases, six were of the HECT (Homologous to E6AP *C*-Terminus) family, and two were of the DCUN1 family (Defective in Cullin Neddylation 1) (Supplementary Table S1A). KEGG analysis of these 58 E3 ligase genes showed that the pathways for endocytosis (*p* = 1.1 × 10^−4^), transcriptional misregulation in cancer (*p* = 4.1 × 10^−4^), and the p53 signaling pathway (*p* = 9.6 × 10^−4^) were significantly overrepresented.

**Figure 5:**
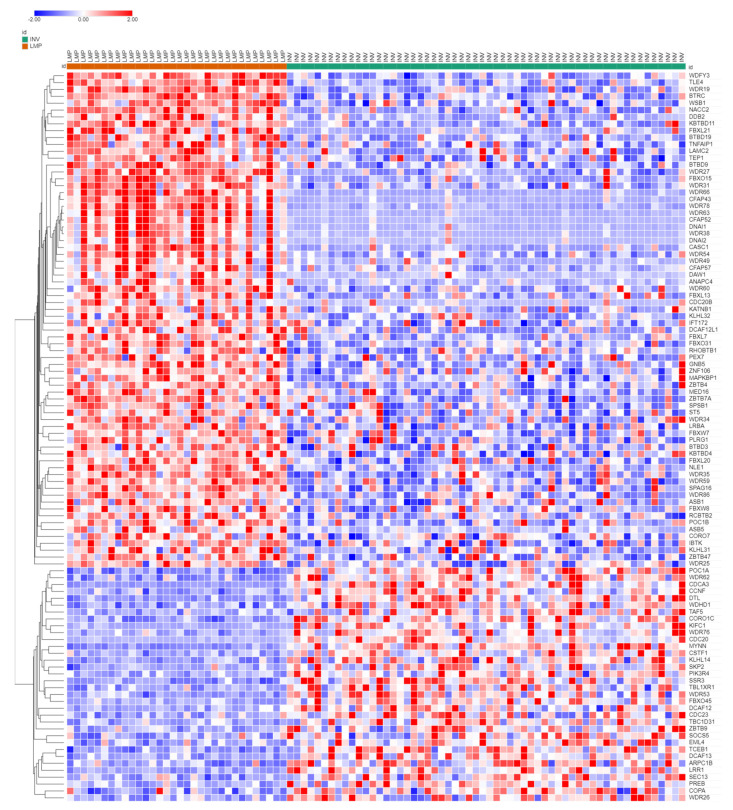
Heatmap and cluster analysis of genes encoding E3 ligase adaptors LMP vs. HGSOC (data from the R2 Genomics Anglesio dataset, 106 genes were significantly different at *p* < 0.001).

Figure 5 illustrates the expression of genes for ubiquitin ligase adaptors. Since ubiquitin ligase adaptors contribute to substrate specificity, they may be more important than ubiquitin ligases as therapeutic targets. KEGG analysis of the 106 genes encoding E3 ligase adaptors showed that two pathways were significantly overrepresented among these genes: the oocyte meiosis pathway (*p* = 1.4 × 10^−5^) and the cell cycle pathway (*p* = 9.7 × 10^−5^). Sixty-five of these genes were identified as belonging to the WDR family of genes. According to the UUCD database, all of the genes depicted in Figure 5 code for E3 Cullin Ring adaptors. Several of these were associated with APC/c. These data suggest that Cullin Ring adaptors may be of importance in regulating the proliferation of HGSOC cells. The role of Cullin Ring ligases (CRLs) in cancer has been reviewed by Jang et al. [62]. Fouad and colleagues discussed targeting CRLs as a therapeutic adjunct to radiation treatment [63]. Carlucci and Angiolella (2015) reviewed the role of CRLs in EOC cells and pointed out that data on the role of specific CRLs in EOC are needed [64].

The expression of genes for ubiquitin ligase adaptors were also grouped into two main clusters (Figure 5). This raised the question whether the transcription of numerous genes encoding for E3 ligases and their adaptors were coregulated. Another explanation would be coregulation of epigenetic variations in E3 ligases and their adaptors. Neddylation would be expected to stimulate the activity of CRLs, but the extent to which NEDD8 modulates transcription factors for CRLs has not been determined.

#### 3.3.2. Neddylation and Cullin Ring Ubiquitin Ligases in EOC

Neddylation stimulates the activity of many CRLs, including those that regulate the cell cycle [65]. One E3 ligase gene, DCUN1D1 (Defective in Cullin Neddylation 1D1, also known as DCNL1), codes for a protein that contributes to the neddylation of CRLs [41]. As such, it could regulate the activity of other E3 ligases. *DCUN1D1* expression was elevated in the HGSOC group (F = 51.947, *p* = 1.86 × 10^−10^). It has been suggested by several research groups that overexpression of the neddylation pathway leads to the activation of many CRLs and the degradation of a number of substrates that act as tumor suppressors [66,67,68,69,70]. An inhibitor of NEDD8 activation, MLN4924 (Figure 3), is undergoing clinical trials as a cancer therapeutic agent [71]. It has been reported to enhance the efficacy of cisplatin use in EOC in mice [72]. We suggest that HGSOC should be added to the list of human clinical trials for this inhibitor, until such time as more specific inhibitors are available.

The removal of the NEDD8 protein from CRLs, deneddylation, is regulated by the COP9 signalosome [73]. The signalosome can therefore deactivate many Cullin Ring ligases. A selective inhibitor of the signalosome, CSN5i-3, has been reported as a potential cancer therapeutic agent [73]. In the Anglesio dataset, the expression of CSN5 (also known as COPS5), which codes for the catalytic subunit of the signalosome, was significantly elevated in the HGSOC cluster (F = 24.893, *p* = 3.03 × 10^−8^). The data suggest the investigation of CSN5i-3 as a therapeutic agent in HGSOC. Expression of Cullin-associated and neddylation-dissociated 1 (*CAND1)*, which codes for another regulator of CRLs, was increased in the HGSOC group (F = 26.980, *p* = 1.31 × 10^−6^). CAND1 is an inhibitory assembly factor for CRLs and binds to deneddylated CRLs [74]. CAND1 has been proposed as a therapeutic target in liver cancer [75]; it should be investigated further in EOC.

#### 3.3.3. The Cullin4 DCX/DWD E3 Subfamily in EOC

Of the 106 differentially expressed E3 CRL adaptors in the Anglesio dataset (at *p* < 0.001), 64 were of the E3 adaptor/Cullin RING/DCX/DWD subfamily (Supplemental Table S1B,C). This subfamily of CRL adaptors uses the DDB1 protein to bind a variety of substrate receptor proteins to Cullin 4 E3 ligases [76,77]. The studies of Higa et al., and Jackson and Xiong showed that these ligases use WDR proteins as substrate adaptors for DDB1-CUL4 E3 ligase complexes [78,79]. According to the UUCD database, 261 genes encoding CRLs of the DCX/DWD family have been identified; as many as 90 have been reported to bind to DDB1. The Cullin 4 CRLs have been reported to contribute to regulation of development, regulation of the cell cycle, regulation of DNA repair, and control of gene transcription [80]. Recently, Bungsy et al. [81] and Lepage et al. [82] assessed the impact of reduced expression of the SKP1-CUL1-F-box protein (SCF) E3 ubiquitin ligase complex members on chromosome instability in immortalized fallopian tube secretory epithelial cells. The SCF complex is composed of four protein subunits, three of which are invariable core components (RING box protein 1 (RBX1), S-phase kinase associated protein 1 (SKP1), and Cullin 1 (CUL1)) and a variable F-box protein that confers substrate specificity [83]. Heterozygous loss of *SKP1*, *RBX1*, and *CUL1* is common in HGSOC patient samples [84]. Models of reduced expression and heterozygous loss of *SKP1*, *RBX1*, or *CUL1* in these HGSOC precursor cells contributed to elevated Cyclin E1 levels and an increase in chromosome instability. The loss of chromosome stability is an early event in HGSOC, and these results indicate that disruption of SCF activity may be an early contributor to HGSOC pathogenesis.

The 64 CRLs of the DCX/DWD family that were differentially expressed in the Anglesio dataset were all of the WDR family of proteins, according to the classification of the Human Genome Nomenclature Committee. These data suggest a significant role for one or more DDB1/CUL4 E3 ligases in EOC. One of these, DDB1/CUL4/CDT2, has been shown to be inhibited by the neddylation inhibitor MLN4924 [85]. The gene encoding the CDT2 (also known as DTL) E3 adaptor component is one of the genes overexpressed several-fold in the HGSOC tumors compared with LMP tumors (Figure 5).

CRLs have been reported to interact with a variety of viruses [86], redirecting their activity. The DDB1-CUL4 E3 ligase has been shown to bind proteins of at least three viruses, the paramyxoviruses, the hepatitis B virus, and HIV-1 [87]. Jackson and Xiong [79] suggested that CUL4 CRLs are often hijacked by viruses. According to these authors, the substrate adaptors of DDB1-CUL4 E3 include DDB2, which has a role in DNA repair.

#### 3.3.4. DDB2 and DNA Repair

*DDB2* codes for a protein that belongs to the DCAF (DDB1 and CUL4 associated factor) family) and acts as a receptor for the CRL4 substrate for global nucleotide excision repair [88,89]. The transcription of this gene is activated by p53 [90]. DDB2 protein levels are reported as low in EOC and it has been suggested to be a tumor suppressor protein [91]. According to the data of Crijns and colleagues, *DDB2* is one of a group of genes whose expression predicts survival in EOC [92]. Low expression is associated with poor outcomes. Expression of DDB2 was significantly reduced (F = 71.069; *p* = 6.10 × 10^−13^) in the HGSOC (INV) group compared with the LMP group, and was one of the genes in the overrepresented p53 KEGG pathway (Table 1) distinguishing HGSOC from LMP. Stimulating DDB2 expression has been suggested as a therapeutic strategy in EOC patients with recurrent disease [91].

### 3.4. BRCA1 Expression in EOC

*BRCA1* is the most well-known of the genes whose mutations have been identified as predisposing to ovarian and breast cancer [93]. Jazaeri et al. were able to distinguish ovarian tumors with *BRCA1* mutations from those with *BRCA2* mutations using a cDNA microarray [94]. BRCA1, together with BARD1, forms a complex that acts as a ubiquitin ligase [3]. The ubiquitin ligase activity of BRCA1 is considered to be important as a suppressor of breast and ovarian cancer [3]. Approximately 15% of women with HGSOC were reported as carrying a *BRCA1* or *BRCA2* mutation [95]. *BRCA1* is located on chromosome 17; it has been reported that loss of a copy of chromosome 17 is a frequent event in the development of HGSOC [96,97]. However, in the Anglesio dataset, the expression of *BRCA1* was modestly elevated in the HGSOC group compared with the LMP group (F = 5.62, *p* = 0.02). Since the Anglesio dataset did not list BRCA mutation status, we cannot relate *BRCA1* transcription to *BRCA1* mutation status in this dataset. BARD1 expression was also elevated in the HGSOC group. Differential expression of *BARD1* was much more significant (F = 33.961, *p* = 9.08 × 10^−^^8^) than that of *BRCA1*. BARD1 is one of the substrates of the APC/c-CDC20 complex and is discussed in this context below.

### 3.5. CDC20 and the APC/c Complex in Cell Division

**Figure 6:**
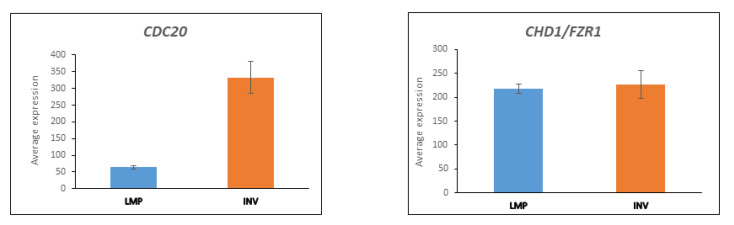
Expression of *CDC20* and *FZR1/CDH1* in LMP and HGSOC (INV) groups from the Anglesio dataset (*CDC20* F = 17.738, *p* = 6.11 × 10^−5^; *FZR1/CDH1* F = 0.057, *p* = 0.81).

The APC/c E3 ligase complex is associated with the two ubiquitin E2 conjugases, UBE2S and UBE2C [98]. APC/c, together with its E3 adaptors Cell Division Cycle 20 (CDC20) and Chromodomain Helicase DNA Binding Proteon 1 (CDH1), controls the degradation of cyclins necessary for transition through the cell cycle [99]. CDC20 has been described as a tumor promotor, while CHD1 is a tumor suppressor [6]. *CDC20* gene expression was elevated (by greater than five-fold) in the HGSOC samples relative to LMP (Figure 6). Expression of the gene encoding CDH1, *FZR1* (not to be confused with the gene *CDH1* encoding cadherin 1), was not significantly different between the LMP and HGSOC groups. The expression data show that the ratio of transcription of the two major E3 ligase adaptors for the APC/c E3 ligase complex is substantially increased in favor of *CDC20* expression in the HGSOC group (Figure 6). APC/c-CDH1 degrades substrates after anaphase, including CDC20. CDH1 is required for APC/c activity after anaphase to the G1–S transition of the cell cycle [100] and is essential in maintaining genomic integrity [101,102].

CDC20 activates the E3 ligase complex APC/c in early and mid-mitosis (during the prophase through to anaphase) and degrades substrates during this part of the cycle, while CDH1 acts later in the cell cycle [103,104]. APC/c-CDC20 initiates chromosome segregation and mitotic exit [105]. The APC/c complex is a major regulator of transcription patterns during the cell cycle [106]. CDC20 overexpression has been associated with poor prognosis in HGSOC [107,108] and in breast cancer [109]. CDC20 has been promoted as a therapeutic target for cancer [110,111,112]. The Anglesio data support the view that CDC20 inhibitors should be tested as therapeutic agents in HGSOC. Gene expression of *PTTG1* and *CCNB1,* which code for the CDC20 substrates securin and Cyclin B1, were elevated in the HGSOC group compared with the LMP group, by 5.12-fold and 3.2-fold, respectively. The Anglesio data confirm the earlier data in Bonome et al.’s study in 2005, in which the expression of the genes *CDC20*, *PTTG1,* and *CCNB1* (encoding cyclin B1) were found to be elevated in HGSOC compared with LMP tumors [108]. During the G2 to M transition, cyclin B is normally protected from destruction by the APC/c-CDC20 complex by an inhibitor of CDC20, MAD2 (also known as MAD2L1) [113]. The expression of the gene encoding the MAD2L1 protein was elevated by greater than two-fold in the HGSOC samples (F = 31.149, *p* = 2.61 × 10^−7^). The expression of CCNB1 was also increased (by greater than three-fold) in the HGSOC group (F = 67.270; *p* = 1.79 × 10^−12^). Collectively, these data provide strong evidence for dysregulation of the APC/c complex in HGSOC.

#### 3.5.1. Substrates of APC/c Are Differentially Expressed between LMP and HGSOC

The substrates of APC/c associated with the cell cycle have been well documented [6,104,114]. Table 4 lists the substrates of APC/c that are differentially expressed between LMP and HGSOC tumors in the Anglesio dataset at various phases of the cell cycle. The expression of all the genes in Table 4 was significantly increased, except for ID2 (Inhibitor of DNA binding 2), whose expression was decreased (F = 18.156, *p* = 5.05 × 10^−9^). ID2 is a regulator of transcription; it is described in GeneCards as a transcriptional misregulator in cancer. The cell cycle-associated substrates of Table 4 include several E3 ligases. These data provide further evidence for dysregulation of the APC/c complex in EOC. The fact that the APC/c complex controls the re-initiation of transcription after mitosis [106] is consistent with the view that the APC/c complex is a key factor in EOC proliferation.

#### 3.5.2. *UBE2C* Expression in LMP and HGSOC

The ubiquitin conjugase UBE2C contributes to the timing of APC/c activity and cell cycle kinases during the cell cycle [92]. CDH1-dependent degradation of UBE2C (UBCH10) by the proteasome results in the accumulation of cyclin A [99]; accumulation of cyclin A, in turn, inactivates APC/c prior to entry of the cell into the S phase [115]. As shown in Table 2 and Table 4 and in the literature, transcription of *UBE2C* and its protein have been shown to be increased in HGSOC compared with LMP tumors [44,45,46,47,116] and UBE2C has been reported as a potential therapeutic target in EOC [43].

In the Anglesio dataset, there is a very high statistical correlation between the expression of *UBE2C* and *AURKA* (*r* = 0.864, *p* = 6.48 × 10^−28^; *r* = 0.839, *p* = 1.05 × 10^−76^). These data suggest the possibility of a common regulatory factor for these two genes. Both of these genes are located on the q arm of chromosome 20. Amplification of genes in this region of chromosome 20 in EOC was noted by Tanner et al. [117]. Jazaeri et al. (2003) suggested a model in which the overexpression of several genes (including *AURKA* and *UBE2C*) located in the 20q13 region of chromosome 20 interfered with centrosome function and mitotic checkpoint control [118], and led to malignant transformation.

#### 3.5.3. AURKA and BARD1 in LMP and HGSOC

*AURKA* and *BARD1* encode proteins (Aurora Kinase A and BRCA1-Associated Ring Domain 1) that have an E3 ligase domain and are substrates of the APC/c E3 ligase complex. The expression of both is significantly greater in the HGSOC group than in the LMP group (Figure 4 and Table 4). *AURKA* is overexpressed in several cancers, including EOC, while in vitro knockdown inhibits proliferation [119]. BARD1 has ben reported to have a tumor suppressor function, while a *BARD1* mutation can increase the risk of EOC [120]. The expression of BARD1 was significantly elevated in the HGSOC group compared with the LMP group (F = 33.96, *p* = 9.08 × 10^−8^) (Figure 4). As noted above, heterodimers of BRCA1 and BARD1 form an E3 complex which contributes to maintenance of genomic stability [3,121,122], and a mutation of *BRCA1* can inactivate the BRCA1/BARD1 complex [3].

Identifying the substrates of the BRCA1/BARD1 complex may clarify the role of this E3 ligase complex in cancer. It can be localized to the centrosome and limits the duplication of centrosomes. Starita and Parvin discussed the ways in which the BRCA1/BARD1 complex regulates centrosome number and chromosome stability [123], including the ubiquitination of gamma tubulin and Nucleophosmin 1 (NPM1) [108,123,124]. RBBP8 (retinoblastoma-binding protein) is a BRCA1/BARD1 substrate that contributes to the G2/M DNA damage checkpoint [125,126,127,128].

The BRCA1/BARD1 complex can be inactivated by platinum-based anticancer drugs [129]. Inhibition of its E3 ligase activity improves the sensitivity to platinum-based therapeutic agents.

**Figure 7:**
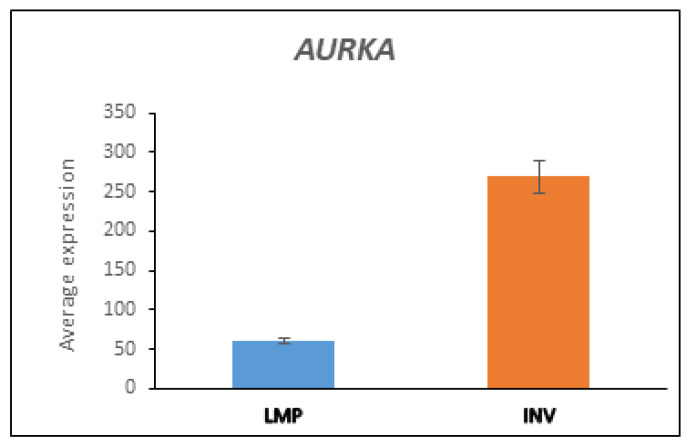
Expression of AURKA in LMP and HGSOC (INV) groups from the Anglesio dataset (F = 56.730, *p* = 4.14 × 10^−11^).

AURKA is essential for the progression of meiosis and proper spindle formation [130]. Several factors regulate *AURKA* transcription and protein stability. AURKA interacts with TPX2 to become fully active [131,132]. The gene encoding TPX2, like that of *AURKA*, is located on chromosome 20q. TPX2 is ubiquitinated by CDC20/CDH1 [133] and is degraded by the proteasome [134]. The E3 ligase CHFR binds to AURKA and reportedly ubiquitinates it prior to proteasomal degradation [135]. AURKA is turned off by Protein Phosphatase 2A [132]. Transcription of *AURKA* is stimulated by MYC [136]. Regulation of the transcription of *AURKA* requires the presence of a cell cycl x 10-dependent element (CDE/CHR) in the *AURKA* promoter [137,138] and the transcription factor E4TF1 [138]. AURKA is a substrate of APC/c that is dependent on the E3 ubiquitin conjugase UBE2S and is overexpressed in several cancers, including EOC [139] (Figure 7). Non-mitotic functions of AURKA include DNA repair, transcription, and cell migration [131]. Do et al. showed that AURKA mediates the migration of EOC cells and recommended the use of AURKA inhibitors in combination with taxane chemotherapy [140].

### 3.6. Expression of Deubiquitinases (DUBs)

Little information is available on the possible role of DUBs in EOC. Table 5 shows the differential expression of genes encoding DUBs. Three of these code for proteins associated with the proteasome: UCHL5, USP14, and PSMD14 [141,142]; as such, they are essential components of the UPS. *EIF3F* codes for a protein that is a translation initiation factor as well as a DUB. Its expression was reduced in HGSOC (INV) compared with LMP tumors. The expression of several other genes for the EIF3 translation initiation complex was also reduced. This suggests that regulation of the initiation of translation of proteins may differ between LMP and HGSOC. The DUB Sentrin-specific protease 2 (SENP) has been reported to reduce the sensitivity of EOC cells to cisplatin [143], and its expression was higher in HGSOC compared with LMP. *OTUD4* codes for a DUB, OTU deubiquitinase 4, which plays a role in DNA damage repair. Expression is lower in HGSOC cancers than in LMP cancers; this is consistent with reports that this gene is downregulated in various cancers [144]. Ubiquitin specific peptidase (USP1) is a DUB whose substrate is FANCD2 (Figure 2) [145]. USP18, while classified as a DUB, removes the ubiquitin-like protein ISG15 from proteins, rather than removing ubiquitin. As such, it is a regulator of the interferon component of the immune response [146]. USP22 is a histone H2B DUB (H2BK120ub1) [147,148,149,150], and H2BK120ub1 deubiquitination is required for DNA double-stranded break repair [151,152]. *USP22* was depressed in the HGSOC group. Expression of USP22 was lower in the HGSOC group compared with the LMP group.

### 3.7. Transcriptome of Proteasome Subunits

The expression of a number of genes coding for proteasome subunits (Table 6) was significantly higher in the HGSOC cluster (16 genes at *p* < 0.001; 25 genes at *p* < 0.01). These data are consistent with reports that increased proteasome activity is observed in various tumors [153,154]. Proteasome inhibitors reportedly stimulate apoptosis in EOC cells [155] and may contribute to a therapeutic approach to EOC [156]. Motosugi and Murata reviewed the regulation of proteasome expression [157]. A transcription factor, NRF1, increases the expression of all proteasome subunits and leads to proteasome synthesis [158,159]. The DDI2 protein is necessary to activate NRF1 [160,161]. Degradation of the NRF1 protein was reported to be regulated by the ubiquitin ligase Hrd1 and the E3 complex SCF/β-TrCP [162]. Targeting NRF1 could provide a potential therapeutic target by reducing the subunits necessary for the degradative activity of the proteasome.

### 3.8. Conclusions

Our analysis of the Anglesio dataset shows that transcription of genes for major components of the ubiquitin proteasome pathway are significantly different, at a high level of statistical significance, between LMP and HGSOC tumors. These proteins include E2 conjugases, E3 ligases, adaptors for E3 ligase complexes, DUBs, and proteasome subunits. Although the mechanisms by which the UPS contributes to HGSOC have not been determined, the current study suggests that the role that the UPS plays in the Fanconi anemia pathway and in the cell cycle pathway may be significant in the progression of EOC. While a great deal of research would need to be conducted to validate the role that inhibition or overexpression of UPS genes may play in LMP and HGSOC pathogenesis, the proteins encoded by these genes may be considered as potential therapeutic targets for the treatment of HGSOC patients.

## Figures and Tables

**Table 1:** Top KEGG pathways distinguishing LMP from HGSOC (INV), including genes for the ubiquitin proteasome pathway.

Overrepresented KEGG Pathway *	Number of Genes	*p*-Value for Pathway	Genes Coding for Components of the Ubiquitin Proteasome Pathway	
Cell cycle	49	1.1 × 10^−11^	*ANAPC4* ^3^, *CDC20* ^3^, *SKP2* ^3^	LMP > INV INV > LMP INV > LMP
DNA replication	19	4.1 × 10^−8^		
p53 signaling	28	1.6 × 10^−7^	*DDB2* ^3^, *MDM2* ^2^, *RFWD2* ^3^	LMP > INV LMP > INV INV > LMP
Huntington’s disease	54	3.06 × 10^−6^	*DNAI1*^3^, *DNAI2* ^3^,	LMP > INV LMP > INV
Fanconi anemia	45	4.6 × 10^−^^4^	*USP1* ^4^, *UBE2T* ^1^	INV > LMP INV > LMP

* Anglesio data (*N* = 90) LMP vs. HGSOC (INV) cluster, log 2 transformation of the top genes by ANOVA at *p* < 0.0001. ^1^ ubiquitin conjugase. ^2^ ubiquitin ligase. ^3^ ubiquitin ligase adaptor. ^4^ deubiquitinase.

**Table 2:** Differential expression of genes encoding ubiquitin conjugases in LMP vs. HGSOC.

Gene	LMP (Means ± se)	HGSOC (Means ± se)	F	*p*
	*N* = 32	*N* = 58		
*UBE2T*	57.42 ± 5.11	278.59 ± 19.41	69.806	8.70 × 10^−13^
*UBE2C*	101.08 ± 6.31	560.62 ± 57.31	35.172	5.81 × 10^−8^
*UBE2W*	133.59 ± 4.89	226.82 ± 9.81	46.139	1.25 × 10^−9^
*UBE2L6*	461.86 ± 23.54	967.39 ± 60.35	36.832	3.17 × 10^−8^
*UBE2K*	318.68 ± 7.98	498.06 ± 22.97	32.321	1.67 × 10^−7^
*UBE2S*	117.97 ± 11.14	345.04 ± 34.57	22.971	6.63 × 10^−6^

**Table 3:** Differential expression of FA genes in Anglesio dataset.

FA Component Gene	LMP Means ± se	HGSOC (INV) Means ± se	F	*p*
*UBE2T*	57.42 ± 5.11	278.59 ± 19.41	69.806	8.70 × 10^−13^
RAD51 *	31.12 ± 2.36	93.94 ± 6.44	50.158	3.32 × 10^−10^
*BRCA2* *	18.12 ± 1.32	47.57 ± 3.20	44.357	2.28 × 10^−9^
*FANCD2*	55.21 ± 3.07	153.36 ± 11.46	39.392	1.27 × 10^−8^
*FANCA*	29.68 ± 2.13	64.47 ± 4.31	33.334	1.15 × 10^−7^
*FANCG*	125.63 ± 4.13	189.16 ± 7.99	32.145	1.78 × 10^−7^
*FANCI*	62.02 ± 4.56	185.60 ± 16.63	29.659	4.62 × 10^−7^
*FANCF*	211.81 ± 8.02	150.65 ± 7.13	29.293	5.31 × 10^−7^
*BRIP1* *	36.78 ± 2.53	105.43 ± 11.57	19.041	3.47 × 10^−5^
*FANCB*	3.04 ± 0.60	8.84 ± 1.04	15.45	1.68 × 10^−4^
*MAD2L2*	104.18 ± 5.53	152.97 ± 8.80	15.087	1.98 × 10^−4^
*FANCC*	45.02 ± 1.91	57.64 ± 2.59	11.231	1.19 × 10^−3^
*RFWD3*	46.58 ± 2.83	64.12 ± 3.80	10.013	2.14 × 10^−3^
*XRCC2* *	97.42 ± 3.97	114.13 ± 4.22	6.801	1.10 × 10^−2^
*PALB2*	146.16 ± 5.05	167.84 ± 5.99	5.932	1.70 × 10^−2^
*BRCA1* *	51.21 ± 3.50	75.88 ± 7.46	5.62	2.00 × 10^−2^

* Genes identified as pathogenic or likely pathogenic and associated with increased lifetime risk of HGSOC [29].

**Table 4:** Differential expression of APC/c substrates between LMP and HGSOC (INV) *.

Prometaphase	Metaphase	Anaphase–Telophase	G1 Phase
NEK2A	CYCLIN B	AURKA	CDC25A
CYCLIN A2	SECURIN	AURKB	SKP2
		CDC20	GEMININ
		PLK1	CDC6
		TPX2	UBE2C
		HEC1	TK1
		BARD1	RRM2
		HMMR	FOXM1
		HURP	ORC1
		NUSAP	ID2
		GEMININ	CDCA3
		ANLN	CCNB1
		PRC1	CLSPN
		SGO1	EMI1

* Gene expression levels for these proteins were all significantly increased at *p* < 0.0001, except for *ID2*, which was decreased.

**Table 5:** Differential expression of genes coding for deubiquitinases.

	LMP (Means ± S.E.)	HGSOC (INV) (Means ± S.E.)	F	*p*
*UCHL5*	132.01 ± 4.26	213.58 ± 7.37	61.097	1.10 × 10^−11^
*USP40*	236.20 ± 10.03	146.94 ± 6.45	60.862	1.18 × 10^−11^
*EIF3F*	3205.01 ± 113.3	2174.67 ± 89.43	49.178	4.57 × 10^−10^
*PSMD14*	650.33 ± 19.43	981.20 ± 35.20	44.434	2.22 × 10^−9^
*SENP2*	134.95 ± 3.55	216.18 ± 9.20	40.930	7.40 × 10^−9^
*USP53*	603.97 ± 60.66	244.64 ± 29.83	35.550	5.06 × 10^−8^
*COPS5*	578.00 ± 22.98	793.79 ± 29.47	24.893	3.03 × 10^−8^
*UFSP2*	275.43 ± 9.83	203.58 ± 8.32	28.872	6.26 × 10^−7^
*OTUD4*	476.54 ± 11.25	373.88 ± 15.00	22.001	9.91 × 10^−8^
*COX8A*	1488.84 ± 52.18	2044 ± 87.17	20.127	2.18 × 10^−5^
*USP1*	699.23 ± 20.99	1102.74 ± 62.22	22.325	8.66 × 10^−6^
*USP18*	58.20 ± 4.38	174.79 ± 17.73	23.319	5.75 × 10^−6^
*USP43*	42.40 ± 2.62	28.57 ± 2.62	30.408	3.46 × 10^−7^
*USP22*	731.28 ± 24.50	558.52 ± 20.31	27.635	1.01 × 10^−6^
*USP39*	106.97 ± 3.81	146.08 ± 5.86	21.693	1.13 × 10^−5^
*FAM63A*	374.76 ± 19.69	287.18 ± 12.70	15.171	1.91 × 10^−4^
*USP14*	560.93 ± 13.07	808.09 ± 38.81	21.528	1.21 × 10^−5^
USP47	381.45 ± 12.84	314.94 ± 11.83	12.828	5.59 × 10^−4^

All differences in expression between the two groups are significantly different at *p* < 0.001.

**Table 6:** Increased expression of genes for proteasome subunits in HGSOC.

Proteasome Gene	Chromosome Location	LMP (Means ± S.E.)	HGSOC (INV) (Means ± S.E.)	F Value	*p* Value
*PSMD2*	3	554.91 ± 17.11	993.61 ± 35.51	77.929	9.29 × 10^−14^
*PSME4*	2	233.77 ± 10.04	409.82 ± 17.64	49.812	3.71 × 10^−10^
*PSMD1*	2	385.60 ± 18.30	623.56 ± 25.11	45.516	4.27 × 10^−9^
*PSMD14*	2	650.33 ± 19.43	981.20 ± 35.20	44.434	2.22 × 10^−9^
*PSMC2*	7	954.96 ± 34.12	1502.52 ± 59.53	42.276	4.64 × 10^−9^
*PSMA7*	20	833.01 ± 36.19	1403.95 ± 64.94	38.811	1.56 × 10^−8^
*PSMB2*	1	810.30 ± 24.63	1566.96 ± 106.75	27.144	1.23 × 10^−6^
*PSMD12*	17	275.32 ± 6.80	470.16 ± 32.54	19.430	2.94 × 10^−5^
*PSMB9*	6	198.19 ± 17.51	525.31 ± 54.63	19.095	3.39 × 10^−5^
*PSMB3*	17	924.33 ± 38.58	1252.30 ± 57.68	15.648	1.54 × 10^−4^
*PSMB4*	1	1769.29 ± 34.96	2274.05 ± 93.15	15.465	1.67 × 10^−4^
*PSMA3*	14	931.61 ± 29.36	1177.96 ± 43.63	15.406	1.72 × 10^−4^
*PSMA2*	7	1118.16 ± 44.05	1396.67 ± 46.99	15.283	1.82 × 10^−4^
*PSMD8*	19	625.55 ± 25.73	965.88 ± 63.49	15.033	2.03 × 10^−4^
*PSMC4*	19	246.34 ± 8.33	432.46 ± 36.56	14.008	3.24 × 10^−4^
*PSMD4*	1	887.14 ± 24.65	1175.07 ± 56.63	13.424	4.24 × 10^−4^

## Data Availability

The data referred to in this manuscript are publicly available at the R2 Genomics Analysis and Visualization Platform (http://r2.amc.nl, accessed on 20 January 2021).

## Supplementary Materials

The following are available online at https://www.mdpi.com/article/10.3390/cancers13112659/s1. Table S1: Gene expression of E3 ligases and E3 adaptors in the Anglesio dataset.
